# Remarks on the Use of Quinine in Yellow Fever

**Published:** 1839-11-30

**Authors:** Thomas J. Donoho

**Affiliations:** Clarksville, Tenn.


*Remarks on the Use of Quinine in Yellow Fever.* By Thomas J. Donoho, M. D.

To the Editors of the Medical Examiner.

Gentlemen,—In the Medical Examiner of the 19th ult., is extracted an article from the New Orleans Bee, on the "Use of Quinine in Yellow Fever."

If my memory does not fail me, Dr. Perrine was the first to prescribe heroic doses of quinine, and the impression is on my mind that he published some observations on the subject in the Medical Recorder; probably, just before he went as minister to some of the South American States. I am not able to say in what year it was, but think about 1829, or 30. Be this as it may, the practice is far from being new in the South, and I always understood Dr. Perrine was the originator. It is true the doses were not so large as mentioned in the extract; they were large enough, however, to constitute it the most successful plan of treatment in the intermittents and remittents of the south. From Dr. McPheters, (among the ablest medical men of the south,) I learned that Dr. P. generally administered from twelve to fifteen grains at a dose, (rarely giving as much as twenty grains,) in the absence of febrile action. It was the first thing done. If an important organ seemed to be much implicated, which is not unfrequently the case, where the disease has progressed for several days, the use of the article was deferred, until by suitable remedies it was relieved. In the treatment of malignant intermittents, I have repeatedly known from twenty to sixty grains given with the happiest effect; nor can I understand how any mode of treating this fearful disease, less potent, can be successful. Unless the second chill, or rather an effort at a chill, be arrested, the patient, in eight cases out of ten, will sink in the cold stage. Diffusible stimuli may arrest it, but they do not break up the associated febrile movement—like quinine, they are not febrifuge, and though the patient finally recovers, he is subject to repeated occasional attacks, when scarcely any treatment will effect a cure, until the weather opens in the month of May; or important organs become irretrievably involved, and death the result.

Administered at the onset of the disease, its effects are in truth *anti-febrile*, and none of the objections urged against it, in the latter stages of fever, hold good, when thus given. As yet, inflammation and its consequences have not supervened; there is nothing more than deranged function. This is the time when the beautiful effects of quinine are displayed. In the remitting form of fever, it may be several days before the remission is sufficiently marked to resort to it. I have occasionally given it, however, when there was smart fever, with good effect. There is increase of fever, pulse more frequent, the forehead hot, and looks somewhat as if it were varnished; tongue frequently becomes dryer, and thirst increased. In a few hours the skin relaxes, and is soon bedewed with a gentle moisture. The pulse now is completely of that which we like to see produced by the bark—fuller and slower; it is sometimes hours before the tongue moistens, and the thirst abates. Sleep follows, but I have never attributed it to the sedative properties of the drug, but to the almost sudden freedom from that peculiarly wretched feeling which no one can understand who has not in his own person experienced it.

A residence of four years in the south, a short distance above Natchez, near the river, has afforded me many opportunities to observe the effects of quinine, and a sedative property would be among the last which I should ascribe to it.

Should those observations meet the eye of Dr. McPheters, of Natchez, I flatter myself with the hope, he will give to the public, through the Examiner, some of the fruits of his long experience on the subject. Of one thing I am sure, but few can bring to the task a sounder judgment or more ability.

*Clarksville, Tenn.*, Nov. 12th, 1839.

